# Synthetic Biology: Caught between Property Rights, the Public Domain, and the Commons

**DOI:** 10.1371/journal.pbio.0050058

**Published:** 2007-03-13

**Authors:** Arti Rai, James Boyle

## Abstract

Synthetic biology presents a particularly revealing example of the difficulty of assimilating a new technology into the conceptual limits around existing intellectual property rights.

Novel artificial genetic systems with twelve bases instead of four [1]. Bacteria that can be programmed to take photographs [2] or form visible patterns [3]. Cells that can count the number of times they divide [4]. A live polio virus “created from scratch using mail-order segments of DNA and a viral genome map that is freely available on the Internet” [5]. These are some of the remarkable, and occasionally disturbing, fruits of “synthetic biology,” the attempt to construct life starting at the genetic level. In terms of their scale and ambition, these efforts go beyond traditional recombinant DNA technology. Rather than simply transferring a pre-existing gene from one species to another, synthetic biologists aim to make biology a true engineering discipline.

In the same way that electrical engineers rely on standard capacitors and resistors, or computer programmers rely on modular blocks of code, synthetic biologists wish to create an array of modular biological parts that can be readily synthesized and mixed together in different combinations. The Massachusetts Institute of Technology (MIT) has a Registry of Standard Biological Parts that supports this goal by indexing biological parts that have been built, and offering assembly services to construct new parts, devices, and systems [6]. Systems, devices, parts, and DNA represent descending levels of complexity—systems consist of devices, and devices consist of parts composed of DNA. The idea behind a registry of parts is that these parts can, and should, be recombined in different ways to produce many different types of devices and systems. Although the registry currently contains physical DNA, its developers believe that, as DNA synthesis technology becomes increasingly inexpensive [7], the registry will be composed largely of information and specifications that can be executed in synthesizers just as semiconductor chip designs are executed by fabrication firms.

Synthetic biology has already produced important results, including more accurate AIDS tests and the possibility of unlimited supplies of previously scarce drugs for malaria [8]. Proponents hope to use synthetic organisms to produce not only medically relevant chemicals but also industrial materials, including biofuels such as hydrogen and ethanol [9]. At the same time, synthetic biology has engendered numerous policy concerns. From its inception, commentators have raised issues ranging from bioethical and environmental worries to fears of bioterrorism—indeed, the US Central Intelligence Agency released a report in 2003 called “The Darker Bioweapons Future” that explicitly referred to the dangers posed by the possibility of genetically engineered viruses [10].

There is, however, one area that has been largely unexplored until this point—the relationship of synthetic biology to intellectual property law. Two key issues deserve further attention. First, synthetic biology presents a particularly revealing example of a difficulty that the law has frequently faced over the last 30 years—the assimilation of a new technology into the conceptual limits posed by existing intellectual property rights. There is reason to fear that tendencies in the way that US law has handled software on the one hand and biotechnology on the other could come together in a “perfect storm” that would impede the potential of the technology. Second, synthetic biology raises with remarkable clarity an issue that has seemed of only theoretical interest until now—the tension between different methods of creating “openness.” On the one hand, one standard mechanism for creating openness has involved putting material in the public domain, outside the world of property. On the other, synthetic biology researchers may want to use intellectual property rights to create a “commons,” just as developers of free and open source software use the leverage of software copyrights to impose requirements of openness on future programmers, requirements greater than those attaching to a public domain work. But synthetic biology, unlike software, is not necessarily protected by copyright. Should we rethink the boundary lines between intellectual property and the public domain as a result?

## The Perfect Storm: Flawed Biotech Law Meets Flawed Software Law?

Intellectual property law in the US has already had difficulty incorporating the revolutionary technologies from which synthetic biology draws inspiration—biotechnology and computers. US patent law requires that inventions be “nonobvious” to the ordinary scientist working in the area. Yet, in the area of biotechnology, years after methods for cloning genes have become routine and widely known, the US Court of Appeals for the Federal Circuit continues to treat the gene products of such methods as patentable [11]. By the Federal Circuit's reasoning, what matters is not whether a practicing biologist would find a particular invention obvious, but rather per se rules about nonobviousness developed for chemical inventions in the mid-20th century [12].

While biotechnology has mainly posed difficulties for patent law, computers have posed both copyright and patent problems. Copyright covers original works of expression, explicitly excluding works that are functional. Patent law requires functionality; however, it had traditionally been understood to exclude formulas and algorithms. Thus, software—a machine made of words, a set of algorithmic instructions devoted to a particular function—seemed to fit neither the copyright nor the patent box. It was too functional for copyright, too close to a collection of algorithms and ideas for patent. What's more, certain economic aspects of software, including its high propensity to display “network effects” (increased utility based on increased numbers of users) led scholars to believe that both copyright and patent were ill-suited to encourage innovation without discouraging competition. Several sui generis, or custom-made, intellectual property regimes were proposed as an alternative.

As a result of statements by the US Congress and actions by the courts, software ended up being covered by both copyright and patent in the US—a result that most scholars thought was far from ideal. Court refusals to allow patent examiners to use unwritten information to determine whether a particular patent application is obvious [13] may also have a disproportionate impact on computer-related inventions. Because much knowledge in the field of computer technology is not written down in journal articles, it may be hard for a patent examiner to find specific written references testifying to information that is generally known. Additionally, many scholars have argued that the Federal Circuit allows unduly broad patents to issue in the area of software [14].

The specter of broad patents has already reared its head in the field of synthetic biology. Consider patent 6,774,222, issued by the US Patent and Trademark Office on August 10, 2004 [15]. The patent, issued to the US Department of Health and Human Services (HHS), is entitled “Molecular Computing Elements, Gates and FlipFlops.” This patent covers using the combination of nucleic-acid binding proteins and nucleic acids to set up data storage as well as logic gates that perform basic Boolean algebra. As the patent document notes, the invention could be used not only for computation but also for complex (“digital”) control of gene expression. The broadest claim does not limit itself to any particular set of nuclei-acid binding proteins or nucleic acids. Moreover, the claim uses language that would cover not only the “parts” that performed the Boolean algebra but also any device and system that contained these parts. Such a patent would seem effectively to patent the basic functions of computing when implemented by one likely genetic means. Would such a foundational patent hold up in court?

Given the low nonobviousness threshold that the Federal Circuit has set in the area of biotechnology, there is some possibility that the court would apply a similarly low threshold here. The Federal Circuit's reluctance to allow unwritten knowledge to be used in determining nonobviousness may also impose a low threshold. Thus, even if, at the time the HHS invention was made, individuals working in the field knew that many computing functions could readily be performed using DNA-based “genetic switches,” this unwritten knowledge might not be factored into the nonobviousness determination. Notably, the HHS patent is not unique in its breadth [16,17].

Considerable historical evidence, including evidence from virtually every important industry of the 20th century, suggests that broad patents on foundational research can slow growth in the industry [18]. In the area of computer hardware, the specter of broad patents loomed large in the US until government action forced licensing of the AT&T transistor patent as well as patents obtained by Texas Instruments and Fairchild Instruments on integrated circuits. Fortunately, software was already a robust industry before broad software patents became available. Biotechnology's foundational technologies—monoclonal antibodies and recombinant techniques—either were not patented or were made available widely at reasonable cost. Synthetic biology may be coming of age under different circumstances, at the juncture of two technologies with which the law is already struggling.

To be sure, to the extent that foundational patents are held by universities or government institutions, they may not be asserted aggressively so as to block research. However, in addition to the problem of broad foundational patents, there is the possibility of a plethora of narrower patents (some of which may fall within the scope of the foundational patents). For example, scientists at Boston University have filed patents that claim the use of DNA to produce specific gene regulation mechanisms such as a multi-state oscillator [19–21]. MIT and the company Sangamo have patents on various types of DNA binding proteins. At least in the area of information technology, there is evidence that patent thickets [22] or “anti-commons” [23] create difficulties for subsequent researchers above and beyond those created by foundational patents. (The situation in biotechnology is less clear; compare [24] and [25].) This is because many products in information technology represent combinations of dozens, if not hundreds, of patented parts. Not only does a crowded patent landscape create the possibility of “hold up” by a previously unknown patent holder who emerges only after others have invested large sums of money in the area of the patented invention, but to the extent that patent rights holders rely upon reach-through royalties to secure revenue, standard economic theory predicts that product output by the improver will be suboptimal. Moreover, while firms that work in information technology have sometimes succeeded in pooling patents, particularly patents around industry standards, such efforts have also been stymied by failure on the part of participating firms to disclose relevant patents [26]. In any event, because synthetic biology encompasses not only information technology but also biotechnology, the absence of successful patent pools in the life sciences is cause for concern.

## A Synthetic Biology Commons?

These intellectual property concerns have not gone unnoticed. The MIT scientists involved with the Registry of Standard Biological Parts are sufficiently concerned that they have created the BioBricks Foundation, which might serve to coordinate a synthetic biology “commons.” The idea of a synthetic biology commons draws inspiration, in part, from the prominence of the open source software model as an alternative to proprietary software. Unlike proprietary software developers, open source software producers make their source code freely available for improvement, modification, and redistribution. Certain types of open source licenses also have a “commons-expanding” aspect: these “copyleft” licenses not only make source code freely available, but they also require those who distribute improvements to the source code to make the improvements available on the same terms (see [27], which discusses GNU General Public License and other “copyleft” licenses). Copylefted software relies heavily on the existence of property rights—specifically, copyright in the source code. Because of this copyright, users of the copylefted software necessarily use it subject to the terms of the license.

Synthetic biologists might argue that strings of DNA bases are comparable to source code and that DNA strings could therefore also be covered by copyright. However, software itself fits poorly into copyright's categories. The US Congress indicated a desire that software be covered by copyright, but left it to the courts to work out the method of doing so. As developed by the courts, copyright protection in software is thin—for example, source code is generally protected against verbatim copying. But even with source code, if the code is entirely dictated by functional concerns or has become an industry standard, it may not be protected by copyright at all.

Where does this leave synthetic biology? There are two major obstacles to establishing copyright. First, unlike software, the products of synthetic biology are not discussed as copyrightable subject matter in the US copyright statute. Thus, a court that wished to find that material copyrightable would have to do so by analogy. Second, even if courts were willing to make such an analogy, there are the internal restrictions of US copyright law, which does not cover functional articles or methods of operation, and requires expressive choices. As a matter of legal doctrine, the answer to whether an expressive choice had been made might depend upon the type of synthetic biology involved. For example, the construction of DNA sequences using base pairs that do not exist in nature might allow significant room for expressive choice. Such DNA sequences might be protected by copyright, at least against verbatim copying. However, most synthetic biologists working today, including those at MIT, are working within the confines of the existing genetic code. This code constrains the expressive choices that they make, making copyright protection less likely.

Thus, in the case of synthetic biology, the ability to invoke copyright is by no means clear. An obvious alternative is patents. One example of a patent-based commons is that created by the group Biological Innovation for an Open Society (BIOS). BIOS is using patent protection on a few key plant gene transfer technologies to force licensees to put improvements to those technologies into the commons [28]. Although some have suggested that the BIOS approach could raise concerns about antitrust and patent misuse [29], the concern should be relatively small given BIOS's mission to expand the commons and the relatively permissive, rule-of-reason-based approach taken by contemporary US antitrust law. The more pressing problem for projects like the MIT Registry of Standard Biological Parts—which contains more than 2,000 standardized parts—is expense. A single patent can cost tens of thousands of dollars to secure.

Of course, to the extent that a few broad patents—like the HHS patent noted above—might effectively cover many of the parts in the registry, the patent option becomes more plausible. In this scenario, the registry would essentially be exploiting flaws in the current patent system for commons-expanding purposes. The difficulty would be to identify an area of inventive territory that was quite broad but nonetheless not suggested either by prior broad patents or by information already in the public domain.

Alternatively, the registry might try to attract statements of non-assertion by other patentees, on the model of recent statements by IBM, Sun Microsystems, and other firms, that they will not assert their patents against anyone working on open source software. Indeed, the fact that many synthetic biology patents are currently held by academic and government institutions may make such statements of assertion a real possibility. To the extent that institutions with synthetic biology patents vowed not to assert their patents against academic researchers, such a move would be a salutary development and a comfort to those working on the registry. Non-assertion statements are not, however, a property right. In order to secure a property right, the owners of the MIT registry would need a license with explicit permission to sublicense. Moreover, patents licensed to the registry would have to cover, at least in some fashion, parts that were important for maintaining and expanding the commons.

Another alternative for securing an expanding commons might rely on some kind of contract, such as a “clickwrap” license over the BioBricks Foundation data. This contractual alternative does not require an underlying property right. Instead, the contract simply imposes conditions as part of the price of access. One problem with such contracts is that they bind only those who receive the technology from the entity imposing the terms. Attempts to prevent leakage to those not bound by the terms of the contract can require strict restrictions on information dissemination. For example, for some time the publicly funded International HapMap Project (a database of human genetic variation) used a clickwrap license. This license required users of single nucleotide polymorphism data to refrain from combining it with their own proprietary single nucleotide polymorphism data in order to seek product patents on haplotypes (collections of single nucleotide polymorphisms). In order to prevent leakage of the data outside the confines of this clickwrap license, to those who would then have no obligation to the HapMap commons, the license required those who sought the data to refrain from disseminating it to anyone who had not signed on to the license. Conventional publication of the data was not possible. This condition is no longer imposed because it is believed that the database has reached a sufficient density to be self-sustaining and to defeat subsequent patent claims. But the old requirements indicate one of the difficulties of the clickwrap approach; the comparative weakness of the contractual restraints paradoxically requires extremely broad restrictions on dissemination.

Finally, legislative proposals might create sui generis property rights mechanisms for protecting BioBricks Foundation data. Indeed, the European Union currently has sui generis protection of data. The evidence suggests, however, that strong property rights protection is likely to hinder rather than promote innovation [30]. A recent draft of the proposed “Treaty on Access to Knowledge” offers an alternative sui generis approach: under this approach, member countries would adopt legislation protecting “qualifying open databases” from patents on certain types of improvements for a specified period of time (Article 5–6 of [31]). Various commentators affiliated with the Access to Knowledge proposal have also suggested the possibility of “social patents” legislation: under this approach, a type of patent right could be secured at low or no cost, but it could not be used for exclusionary commercial purposes. Although these sui generis alternatives are quite intriguing, and certainly an improvement over ordinary property rights in databases, securing new legislation is a difficult, uncertain, and slow route. Table 1 summarizes the advantages and disadvantages of a sui generis strategy as well as other strategies.

**Table 1:**
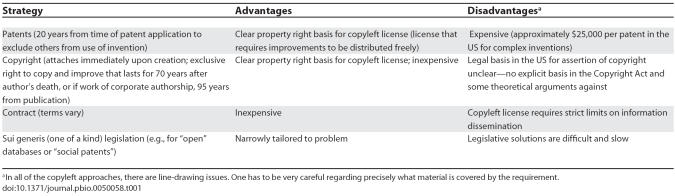
Alternatives for Synthetic Biology

We close with one overarching observation. Copyleft licenses, which lead to the formation of an ever-expanding commons, have worked well—even brilliantly—in the software context. These licenses have produced well-functioning code, and they have also constrained the threat posed by copyright and patent, particularly when such intellectual property could be attached to an incipient industry standard. Would they work as well in synthetic biology? There is reason for some caution. Intellectual property rights are relatively unimportant as incentives at any stage in the production of copyleft software. They are important mainly for the leverage they give to the licensor. But synthetic biology might be different. Though the uses of synthetic biology are by no means limited to biomedicine, at the end of some biological chains of innovation will lie the expensive development and commercialization of a drug. While taking a drug all the way through clinical trials mandated by the US Food and Drug Administration may not cost as much as drug companies claim, it does cost hundreds of millions of dollars. Whether patent rights are the best incentive mechanism for purposes of eliciting pharmaceutical R&D is not a question we can address here. Suffice it to say that our current system of financing pharmaceutical innovation relies heavily on these rights. There is no direct equivalent in the world of free software. If a copyleft condition—however drafted and imposed—did attach to some of synthetic biology's parts, care would have to be taken in the design of the system, lest the result be to make it impossible for that technology to be developed into a patented therapy. The BIOS licenses, which restrict the copyleft condition to improvements on the enabling technology and do not constrain patenting on transgenic plant products, provide an interesting model. But the distinction between enabling technology and product may be easier to make in a situation like that faced by BIOS, where the enabling technology in question has a relatively clear innovation trajectory, both in terms of improvement to the technology itself and in terms of production of end products.

In the meantime, the decision, already implemented, of the MIT Registry of Standard Biological Parts to place its parts into the public domain certainly provides important protection against the threat of patents clogging innovation in the synthetic biology space. Placing parts into the public domain not only makes the parts unpatentable, but it undermines the possibility of patents on trivial improvements. In the end, a public domain strategy comparable to that employed by the public Human Genome Project may not be ideal, but it is certainly a good start.

## Abbreviations

BIOS: Biological Innovation for an Open Society
HHS: US Department of Health and Human Services
MIT: Massachusetts Institute of Technology
